# 2,5-Dimethyl-7,8,9,10-tetra­hydro­cyclo­hepta­[*b*]indol-6(5*H*)-one

**DOI:** 10.1107/S1600536810041772

**Published:** 2010-10-23

**Authors:** R. Archana, E. Yamuna, K. J. Rajendra Prasad, A. Thiruvalluvar, R. J. Butcher

**Affiliations:** a PG Research Department of Physics, Rajah Serfoji Government College (Autonomous), Thanjavur 613 005, Tamilnadu, India; b Department of Chemistry, Bharathiar University, Coimbatore 641 046, Tamilnadu, India; c Department of Chemistry, Howard University, 525 College Street NW, Washington, DC 20059, USA

## Abstract

In the title mol­ecule, C_15_H_17_NO, the dihedral angle between the benzene and pyrrole rings is 1.45 (13)°. The cyclo­heptene ring adopts a slightly distorted boat conformation. In the crystal structure, inter­molecular C—H⋯O hydrogen bonds are found.

## Related literature

For the importance of the indole nucleus, see: Satoshi & Tominari (2001 ▶). For the synthesis of fused cyclo­hept[*b*]indole derivatives, see: Butin *et al.* (2010 ▶); Fujimori & Yamane (1978 ▶); Wahlström *et al.* (2007 ▶). For heteroannulated cyclo­hept[*b*]indole derivatives, see: Kavitha & Prasad (1999 ▶, 2001 ▶). For crystallographic studies of cyclo­hept[*b*]indoles, see: Sridharan *et al.* (2008*a* ▶,*b* ▶, 2009 ▶); Yamuna *et al.* (2010 ▶).
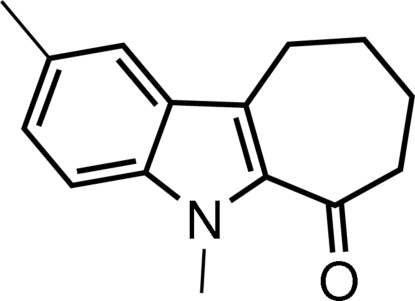


## Experimental

### 

#### Crystal data

C_15_H_17_NO
*M* *_r_* = 227.30
Orthorhombic, 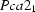

*a* = 15.5889 (3) Å
*b* = 10.5707 (2) Å
*c* = 7.5388 (2) Å
*V* = 1242.29 (5) Å^3^
*Z* = 4
Cu *K*α radiation
μ = 0.59 mm^−1^
*T* = 295 K
0.49 × 0.32 × 0.12 mm

#### Data collection

Oxford Diffraction Xcalibur Ruby Gemini diffractometer
Absorption correction: multi-scan (*CrysAlis PRO*; Oxford Diffraction, 2010 ▶) *T*_min_ = 0.887, *T*_max_ = 1.000
1327 measured reflections
1327 independent reflections
1285 reflections with *I* > 2σ(*I*)

#### Refinement

*R*[*F* ^2^ > 2σ(*F* ^2^)] = 0.045
*wR*(*F* ^2^) = 0.129
*S* = 1.09
1327 reflections
156 parameters
1 restraint
H-atom parameters constrained
Δρ_max_ = 0.17 e Å^−3^
Δρ_min_ = −0.16 e Å^−3^

### 

Data collection: *CrysAlis PRO* (Oxford Diffraction, 2010 ▶); cell refinement: *CrysAlis PRO*; data reduction: *CrysAlis PRO*; program(s) used to solve structure: *SHELXS97* (Sheldrick, 2008 ▶); program(s) used to refine structure: *SHELXL97* (Sheldrick, 2008 ▶); molecular graphics: *ORTEP-3* (Farrugia, 1997 ▶) and *PLATON* (Spek, 2009 ▶); software used to prepare material for publication: *PLATON*.

## Supplementary Material

Crystal structure: contains datablocks global, I. DOI: 10.1107/S1600536810041772/hg2724sup1.cif

Structure factors: contains datablocks I. DOI: 10.1107/S1600536810041772/hg2724Isup2.hkl

Additional supplementary materials:  crystallographic information; 3D view; checkCIF report

## Figures and Tables

**Table 1:** Hydrogen-bond geometry (Å, °)

*D*—H⋯*A*	*D*—H	H⋯*A*	*D*⋯*A*	*D*—H⋯*A*
C10—H10*B*⋯O6^i^	0.97	2.59	3.550 (3)	168

Symmetry code: (i) 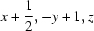.

**Fig. 1.**
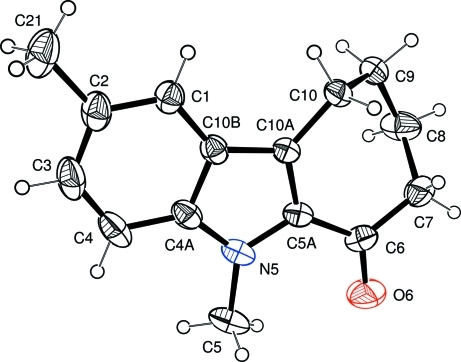
The molecular structure of the title compound, showing the atom-numbering scheme and displacement ellipsoids drawn at the 30% probability level. H atoms are shown as small spheres of arbitrary radius.

**Fig. 2.**
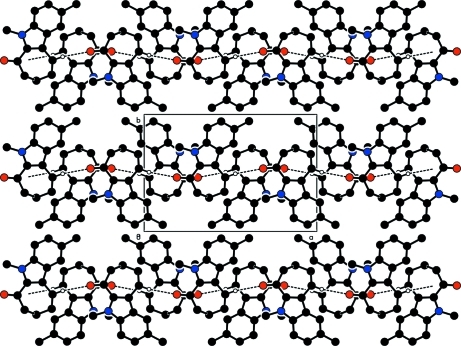
The molecular packing of the title compound, viewed down the c axis. Dashed lines indicate hydrogen bonds. H atoms not involved in hydrogen bonding have been omitted.

## supplementary crystallographic information

### Comment

Since the indole nucleus is present in a large number of naturally occurring as well as biologically active molecules, indole derivatives are of considerable contemporary interest and importance (Satoshi & Tominari, 2001). Due to the importance of these compounds, several fused cyclohept[*b*]indole derivatives have been synthesized (Butin *et al.*, 2010); Fujimori & Yamane, 1978); Wahlström *et al.*, 2007)). In our laboratory 7,8,9,10-tetrahydrocyclohepta[*b*]indol-6(5*H*)-one was used as a synthon to derive various heteroannulated cyclohept[*b*]indole derivatives (Kavitha & Prasad 1999, 2001). Recently we have reported crystallographic studies for some cyclohept[*b*]indoles from our laboratory (Sridharan *et al.*, 2008*a*,*b*, 2009); Yamuna *et al.*, 2010). For optimal drug design, knowledge of the exact geometry and shape of the molecule is essential and thus we decided to subject the compounds synthesized to single-crystal X-ray diffraction studies.

The molecular structure of the title compound, with atomic numbering scheme, is shown in Fig. 1. In the title molecule, C_15_H_17_NO, the dihedral angle between the benzene and pyrrole rings is 1.45 (13)°. The cycloheptene ring adopts a slightly distorted boat conformation. In the crystal structure intermolecular C—H···O hydrogen bonds are found (Table 1, Fig. 2).

### Experimental

To a solution of 2-methyl-7,8,9,10-tetrahydrocyclohepta[*b*]indol-6(5*H*)-one (0.213 g, 0.001 mol) in 5 ml acetone added powdered KOH (0.280 g, 0.005 mol) in ice cold condition. After few minutes methyl iodide (0.13 ml, 0.002 mol) was added drop by drop with vigorous stirring and the reaction mixture was stirrired for 15 min at room temperature. Benzene was added to the reaction mixture and insoluble materials are removed by filtration. The benzene solution was washed with saturated NaCl solution, dried by using Na_2_SO_4_ and evaporation yielded the title compound (0.204 g, 90%). This was recrystallized from benzene and ethyl acetate mixture.

### Refinement

Owing to the absence of any anamalous scatterers in the molecule, the Friedel pairs were merged. The absolute structure in the present model have been chosen arbitrarily. H atoms were positioned geometrically and allowed to ride on their parent atoms, with C—H = 0.93 - 0.97 Å and *U*_iso_(H) = 1.2 - 1.5 times *U*_eq_(C).

### Crystal data

**Table:**

C_15_H_17_NO	*D*_x_ = 1.215 Mg m^−^^3^
*M**_r_* = 227.30	Melting point: 346 K
Orthorhombic, *P**c**a*2_1_	Cu *K*α radiation, λ = 1.54184 Å
Hall symbol: P 2c -2ac	Cell parameters from 2494 reflections
*a* = 15.5889 (3) Å	θ = 5.1–73.7°
*b* = 10.5707 (2) Å	µ = 0.59 mm^−^^1^
*c* = 7.5388 (2) Å	*T* = 295 K
*V* = 1242.29 (5) Å^3^	Plate, pale yellow-orange
*Z* = 4	0.49 × 0.32 × 0.12 mm
*F*(000) = 488	

### Data collection

**Table:**

Oxford Diffraction Xcalibur Ruby Gemini diffractometer	1327 independent reflections
Radiation source: Enhance (Cu) X-ray Source	1285 reflections with *I* > 2σ(*I*)
graphite	*R*_int_ = 0.0000
Detector resolution: 10.5081 pixels mm^-1^	θ_max_ = 73.8°, θ_min_ = 5.1°
ω scans	*h* = 0→19
Absorption correction: multi-scan (*CrysAlis PRO*; Oxford Diffraction, 2010)	*k* = 0→13
*T*_min_ = 0.887, *T*_max_ = 1.000	*l* = 0→9
1327 measured reflections	

### Refinement

**Table:**

Refinement on *F*^2^	Primary atom site location: structure-invariant direct methods
Least-squares matrix: full	Secondary atom site location: difference Fourier map
*R*[*F*^2^ > 2σ(*F*^2^)] = 0.045	Hydrogen site location: inferred from neighbouring sites
*wR*(*F*^2^) = 0.129	H-atom parameters constrained
*S* = 1.09	*w* = 1/[σ^2^(*F*_o_^2^) + (0.097*P*)^2^ + 0.041*P*] where *P* = (*F*_o_^2^ + 2*F*_c_^2^)/3
1327 reflections	(Δ/σ)_max_ = 0.001
156 parameters	Δρ_max_ = 0.17 e Å^−^^3^
1 restraint	Δρ_min_ = −0.16 e Å^−^^3^

### Special details

**Table:**

Geometry. Bond distances, angles *etc*. have been calculated using the rounded fractional coordinates. All su's are estimated from the variances of the (full) variance-covariance matrix. The cell e.s.d.'s are taken into account in the estimation of distances, angles and torsion angles
Refinement. Refinement of *F*^2^ against ALL reflections. The weighted *R*-factor *wR* and goodness of fit *S* are based on *F*^2^, conventional *R*-factors *R* are based on *F*, with *F* set to zero for negative *F*^2^. The threshold expression of *F*^2^ > 2σ(*F*^2^) is used only for calculating *R*-factors(gt) *etc*. and is not relevant to the choice of reflections for refinement. *R*-factors based on *F*^2^ are statistically about twice as large as those based on *F*, and *R*- factors based on ALL data will be even larger.

### Fractional atomic coordinates and isotropic or equivalent isotropic displacement parameters (Å^2^)

**Table:**

	*x*	*y*	*z*	*U*_iso_*/*U*_eq_	
O6	0.18943 (11)	0.4617 (2)	0.5238 (5)	0.1003 (11)	
N5	0.29328 (12)	0.68004 (18)	0.4471 (3)	0.0561 (6)	
C1	0.51734 (15)	0.75136 (19)	0.4664 (3)	0.0534 (6)	
C2	0.52228 (19)	0.8760 (2)	0.4100 (4)	0.0667 (8)	
C3	0.4462 (2)	0.9404 (2)	0.3639 (5)	0.0789 (9)	
C4	0.3667 (2)	0.8856 (2)	0.3734 (4)	0.0738 (9)	
C4A	0.36199 (15)	0.7584 (2)	0.4294 (3)	0.0534 (6)	
C5	0.20448 (16)	0.7199 (3)	0.4212 (5)	0.0757 (9)	
C5A	0.32271 (11)	0.56233 (19)	0.5029 (3)	0.0478 (5)	
C6	0.26683 (13)	0.4538 (2)	0.5353 (3)	0.0573 (6)	
C7	0.30755 (16)	0.3306 (2)	0.5819 (5)	0.0711 (9)	
C8	0.37596 (17)	0.2890 (3)	0.4485 (6)	0.0817 (12)	
C9	0.46482 (15)	0.3391 (2)	0.4821 (4)	0.0614 (7)	
C10	0.46951 (13)	0.46270 (19)	0.5847 (4)	0.0536 (6)	
C10A	0.41137 (11)	0.56610 (16)	0.5207 (3)	0.0434 (5)	
C10B	0.43718 (13)	0.69124 (18)	0.4753 (3)	0.0469 (5)	
C21	0.6075 (3)	0.9427 (3)	0.3943 (6)	0.0945 (13)	
H1	0.56686	0.70786	0.49820	0.0640*	
H3	0.45022	1.02372	0.32532	0.0947*	
H4	0.31752	0.93066	0.34395	0.0886*	
H5A	0.20287	0.79065	0.34142	0.1136*	
H5B	0.18019	0.74410	0.53321	0.1136*	
H5C	0.17200	0.65120	0.37201	0.1136*	
H7A	0.26345	0.26605	0.58845	0.0853*	
H7B	0.33362	0.33765	0.69835	0.0853*	
H8A	0.37840	0.19726	0.44833	0.0980*	
H8B	0.35808	0.31561	0.33110	0.0980*	
H9A	0.49307	0.35096	0.36871	0.0737*	
H9B	0.49689	0.27543	0.54691	0.0737*	
H10A	0.45605	0.44540	0.70798	0.0643*	
H10B	0.52813	0.49334	0.58032	0.0643*	
H21A	0.65287	0.88480	0.42275	0.1418*	
H21B	0.60900	1.01291	0.47511	0.1418*	
H21C	0.61481	0.97296	0.27521	0.1418*	

### Atomic displacement parameters (Å^2^)

**Table:**

	*U*^11^	*U*^22^	*U*^33^	*U*^12^	*U*^13^	*U*^23^
O6	0.0441 (8)	0.0967 (14)	0.160 (3)	−0.0048 (8)	0.0028 (14)	0.0057 (18)
N5	0.0518 (9)	0.0564 (10)	0.0600 (11)	0.0183 (8)	−0.0059 (8)	−0.0087 (9)
C1	0.0627 (11)	0.0451 (9)	0.0524 (12)	−0.0035 (8)	0.0046 (9)	−0.0064 (8)
C2	0.0934 (17)	0.0432 (10)	0.0636 (14)	−0.0093 (10)	0.0126 (13)	−0.0110 (11)
C3	0.122 (2)	0.0374 (9)	0.0772 (18)	−0.0010 (12)	0.0128 (18)	−0.0027 (12)
C4	0.1000 (19)	0.0475 (12)	0.0740 (17)	0.0272 (12)	−0.0020 (14)	−0.0022 (12)
C4A	0.0629 (12)	0.0465 (10)	0.0509 (10)	0.0129 (8)	0.0003 (9)	−0.0069 (9)
C5	0.0588 (13)	0.0851 (17)	0.0833 (17)	0.0332 (13)	−0.0132 (13)	−0.0156 (16)
C5A	0.0438 (9)	0.0511 (10)	0.0485 (9)	0.0080 (7)	0.0005 (8)	−0.0059 (9)
C6	0.0440 (9)	0.0653 (11)	0.0625 (12)	−0.0037 (8)	0.0062 (9)	−0.0094 (11)
C7	0.0565 (11)	0.0587 (12)	0.098 (2)	−0.0114 (10)	0.0112 (13)	0.0050 (15)
C8	0.0641 (13)	0.0681 (14)	0.113 (3)	0.0050 (11)	−0.0031 (16)	−0.0378 (19)
C9	0.0596 (11)	0.0469 (10)	0.0777 (15)	0.0111 (8)	0.0124 (11)	0.0058 (11)
C10	0.0425 (8)	0.0492 (10)	0.0691 (14)	0.0037 (7)	−0.0056 (9)	0.0103 (10)
C10A	0.0429 (8)	0.0430 (9)	0.0444 (9)	0.0042 (7)	−0.0001 (8)	−0.0025 (8)
C10B	0.0567 (10)	0.0404 (9)	0.0436 (9)	0.0062 (7)	0.0024 (8)	−0.0033 (8)
C21	0.120 (3)	0.0646 (15)	0.099 (2)	−0.0374 (17)	0.018 (2)	−0.0114 (17)

### Geometric parameters (Å, °)

**Table:**

O6—C6	1.213 (3)	C10A—C10B	1.424 (3)
N5—C4A	1.361 (3)	C1—H1	0.9300
N5—C5	1.460 (3)	C3—H3	0.9300
N5—C5A	1.391 (3)	C4—H4	0.9300
C1—C2	1.387 (3)	C5—H5A	0.9600
C1—C10B	1.404 (3)	C5—H5B	0.9600
C2—C3	1.411 (4)	C5—H5C	0.9600
C2—C21	1.509 (5)	C7—H7A	0.9700
C3—C4	1.370 (4)	C7—H7B	0.9700
C4—C4A	1.411 (3)	C8—H8A	0.9700
C4A—C10B	1.413 (3)	C8—H8B	0.9700
C5A—C6	1.461 (3)	C9—H9A	0.9700
C5A—C10A	1.389 (2)	C9—H9B	0.9700
C6—C7	1.491 (3)	C10—H10A	0.9700
C7—C8	1.530 (5)	C10—H10B	0.9700
C8—C9	1.505 (4)	C21—H21A	0.9600
C9—C10	1.520 (3)	C21—H21B	0.9600
C10—C10A	1.500 (3)	C21—H21C	0.9600
			
O6···N5	2.878 (3)	H1···O6^viii^	2.6300
O6···C5	2.847 (4)	H3···H8A^vi^	2.3400
O6···H5C	2.3200	H4···C5	2.9000
O6···H7B^i^	2.8100	H4···H5A	2.3200
O6···H8B^ii^	2.8800	H5A···C4	2.7500
O6···H1^iii^	2.6300	H5A···H4	2.3200
O6···H10B^iii^	2.5900	H5B···C4^ii^	3.0600
N5···O6	2.878 (3)	H5B···C4A^ii^	3.0600
C5···O6	2.847 (4)	H5C···O6	2.3200
C5···C5A^i^	3.591 (4)	H5C···C6	2.8400
C5A···C5^ii^	3.591 (4)	H5C···C5A^i^	2.9400
C1···H10A^iv^	2.8800	H5C···C10A^i^	3.0800
C1···H10B	2.8600	H7B···C10	2.6400
C3···H21B^v^	3.0900	H7B···C10A	3.0200
C3···H8A^vi^	2.9800	H7B···H10A	2.2200
C4···H5B^i^	3.0600	H7B···O6^ii^	2.8100
C4···H5A	2.7500	H8A···C3^ix^	2.9800
C4A···H5B^i^	3.0600	H8A···H3^ix^	2.3400
C5···H4	2.9000	H8B···C5A	2.9600
C5A···H8B	2.9600	H8B···O6^i^	2.8800
C5A···H5C^ii^	2.9400	H9A···C10^iv^	2.9700
C6···H5C	2.8400	H9A···H10A^iv^	2.5900
C7···H10A	2.7800	H10A···C7	2.7800
C10···H1	3.0700	H10A···H7B	2.2200
C10···H7B	2.6400	H10A···C1^vii^	2.8800
C10···H9A^vii^	2.9700	H10A···C10B^vii^	2.9900
C10A···H7B	3.0200	H10A···H9A^vii^	2.5900
C10A···H5C^ii^	3.0800	H10B···C1	2.8600
C10B···H10A^iv^	2.9900	H10B···H1	2.4300
H1···C10	3.0700	H10B···O6^viii^	2.5900
H1···H10B	2.4300	H21A···H1	2.3700
H1···H21A	2.3700	H21B···C3^x^	3.0900
			
C4A—N5—C5	123.9 (2)	C4A—C4—H4	121.00
C4A—N5—C5A	108.33 (17)	N5—C5—H5A	109.00
C5—N5—C5A	127.7 (2)	N5—C5—H5B	109.00
C2—C1—C10B	119.6 (2)	N5—C5—H5C	109.00
C1—C2—C3	119.2 (2)	H5A—C5—H5B	109.00
C1—C2—C21	121.1 (3)	H5A—C5—H5C	109.00
C3—C2—C21	119.7 (2)	H5B—C5—H5C	109.00
C2—C3—C4	122.9 (2)	C6—C7—H7A	109.00
C3—C4—C4A	117.8 (2)	C6—C7—H7B	109.00
N5—C4A—C4	130.6 (2)	C8—C7—H7A	109.00
N5—C4A—C10B	108.85 (18)	C8—C7—H7B	109.00
C4—C4A—C10B	120.6 (2)	H7A—C7—H7B	108.00
N5—C5A—C6	123.79 (17)	C7—C8—H8A	108.00
N5—C5A—C10A	109.37 (17)	C7—C8—H8B	108.00
C6—C5A—C10A	126.84 (18)	C9—C8—H8A	108.00
O6—C6—C5A	121.8 (2)	C9—C8—H8B	108.00
O6—C6—C7	120.1 (2)	H8A—C8—H8B	107.00
C5A—C6—C7	118.13 (18)	C8—C9—H9A	108.00
C6—C7—C8	113.1 (3)	C8—C9—H9B	108.00
C7—C8—C9	115.5 (3)	C10—C9—H9A	108.00
C8—C9—C10	115.6 (2)	C10—C9—H9B	108.00
C9—C10—C10A	115.7 (2)	H9A—C9—H9B	107.00
C5A—C10A—C10	127.70 (17)	C9—C10—H10A	108.00
C5A—C10A—C10B	106.53 (16)	C9—C10—H10B	108.00
C10—C10A—C10B	125.70 (16)	C10A—C10—H10A	108.00
C1—C10B—C4A	119.95 (18)	C10A—C10—H10B	108.00
C1—C10B—C10A	133.12 (19)	H10A—C10—H10B	107.00
C4A—C10B—C10A	106.92 (17)	C2—C21—H21A	109.00
C2—C1—H1	120.00	C2—C21—H21B	109.00
C10B—C1—H1	120.00	C2—C21—H21C	109.00
C2—C3—H3	119.00	H21A—C21—H21B	109.00
C4—C3—H3	119.00	H21A—C21—H21C	110.00
C3—C4—H4	121.00	H21B—C21—H21C	109.00
			
C5—N5—C4A—C4	−5.0 (4)	C4—C4A—C10B—C10A	−178.4 (2)
C5—N5—C4A—C10B	176.1 (3)	N5—C5A—C6—O6	−3.8 (4)
C5A—N5—C4A—C4	178.6 (3)	N5—C5A—C6—C7	175.6 (2)
C5A—N5—C4A—C10B	−0.4 (3)	C10A—C5A—C6—O6	176.5 (3)
C4A—N5—C5A—C6	−179.7 (2)	C10A—C5A—C6—C7	−4.1 (4)
C4A—N5—C5A—C10A	0.0 (3)	N5—C5A—C10A—C10	177.3 (2)
C5—N5—C5A—C6	4.0 (4)	N5—C5A—C10A—C10B	0.4 (3)
C5—N5—C5A—C10A	−176.4 (3)	C6—C5A—C10A—C10	−3.1 (4)
C10B—C1—C2—C3	0.5 (4)	C6—C5A—C10A—C10B	−179.9 (2)
C10B—C1—C2—C21	−178.4 (3)	O6—C6—C7—C8	126.1 (3)
C2—C1—C10B—C4A	−0.9 (3)	C5A—C6—C7—C8	−53.3 (3)
C2—C1—C10B—C10A	177.5 (3)	C6—C7—C8—C9	86.9 (3)
C1—C2—C3—C4	0.5 (5)	C7—C8—C9—C10	−25.7 (4)
C21—C2—C3—C4	179.5 (3)	C8—C9—C10—C10A	−48.2 (3)
C2—C3—C4—C4A	−1.0 (5)	C9—C10—C10A—C5A	57.1 (3)
C3—C4—C4A—N5	−178.3 (3)	C9—C10—C10A—C10B	−126.6 (2)
C3—C4—C4A—C10B	0.6 (4)	C5A—C10A—C10B—C1	−179.3 (2)
N5—C4A—C10B—C1	179.5 (2)	C5A—C10A—C10B—C4A	−0.7 (3)
N5—C4A—C10B—C10A	0.6 (3)	C10—C10A—C10B—C1	3.9 (4)
C4—C4A—C10B—C1	0.4 (3)	C10—C10A—C10B—C4A	−177.5 (2)

Symmetry codes: (i) −*x*+1/2, *y*, *z*−1/2; (ii) −*x*+1/2, *y*, *z*+1/2; (iii) *x*−1/2, −*y*+1, *z*; (iv) −*x*+1, −*y*+1, *z*−1/2; (v) −*x*+1, −*y*+2, *z*−1/2; (vi) *x*, *y*+1, *z*; (vii) −*x*+1, −*y*+1, *z*+1/2; (viii) *x*+1/2, −*y*+1, *z*; (ix) *x*, *y*−1, *z*; (x) −*x*+1, −*y*+2, *z*+1/2.

### Hydrogen-bond geometry (Å, °)

**Table:**

*D*—H···*A*	*D*—H	H···*A*	*D*···*A*	*D*—H···*A*
C10—H10B···O6^viii^	0.97	2.59	3.550 (3)	168

Symmetry codes: (viii) *x*+1/2, −*y*+1, *z*.
